# Corneal, Scleral, Choroidal, and Foveal Thickness in Patients with Rheumatoid Arthritis

**DOI:** 10.4274/tjo.36528

**Published:** 2018-12-27

**Authors:** Kelvin Z. Li, Colin S. Tan

**Affiliations:** 1 National Health Group Eye Institute, Tan Tock Seng Hospital, Singapore

**Keywords:** Rheumatoid arthritis, scleral thickness, corneal thickness, choroidal-retinal thickness, optical coherence tomography

## Dear Editor,

We congratulate Gökmen et al.^1^ for their paper evaluating corneal, scleral, choroidal, and foveal thickness in patients with rheumatoid arthritis (RA). While the authors found that female patients with RA had a thinner sclera compared to healthy subjects, there was no difference for corneal, choroidal and foveal thickness.

Assessment of the choroid is important because it provides nutrition to the outer retinal structures and hence plays a role in many chorioretinal diseases. While the authors did not find any statistical difference in choroidal thicknesses between the two groups, those with RA were noted to have consistently thicker choroids in all measurements points except at 3 mm nasally. This trend may be interesting.

The authors obtained the choroidal thickness by averaging measurements taken at seven specific points. However, the choroid is a three-dimensional structure with considerable topographic variation.^2,3^ Measuring the mean choroidal thickness in different regions of the macula by manual segmentation of the choroid-scleral interface may potentially yield interesting findings.

Similarly, this is a potential consideration when assessing retinal thickness. The authors used the central foveal thickness, which was measured manually from the internal limiting membrane to the retinal pigment epithelium at the fovea. An alternative would be to assess the central subfield retinal thickness using the automated segmentation provided by the proprietary software on Optical Coherence Tomography devices.^4^ It has also been shown that the central retinal thickness has less variability than the central point thickness.^5^

In summary, the authors presented interesting findings of a thinner sclera in patients with RA as compared to healthy subjects. The use of choroidal segmentation technique and central retinal thickness may enhance the evaluation of the respective anatomical structures in future studies.
