# Supplementary material for: Corneal, Scleral, Choroidal, and Foveal Thickness in Patients with Rheumatoid Arthritis
Source: Turk J Ophthalmol. 2018 Dec 27;48(6):326–7. doi: 10.4274/tjo.36528 (PMC6330666; doi:10.4274/tjo.36528)
Supplement: Related texts

# Reply: Response from the Authors

**Authors:** Onur Gökmen, Ahmet Akman, Sirel Gür Güngör

## Dear Editor,

We are happy to receive a letter concerning our study about corneal, scleral, choroidal, and foveal thickness in patients with rheumatoid arthritis (RA) and we thank Li and Tan for their positive contributions to our study.

In our study we used the single-slice manual segmentation technique around the fovea; however, rheumatoid arthritis is a systemic disease and systemic diseases may affect not only the macula, but all retinal and choroidal vascular structures. Perhaps in the near future, newly developed devices may enable wide-field automated choroidal measurements, thus providing a better understanding of the topographic variations of the three-dimensional vascular structure of the choroid.

For scleral measurements we used time domain optical coherence tomography (OCT) with a 45-degree temporal gaze at one point, but laser penetration of the sclera was significantly lower than retina or choroid, which affects resolution of the uvea-scleral junctional image. However, using swept source anterior segment OCT provides much better image quality and the combination of ultrasound biomicroscopy at multiple points may yield higher quality images and data.^1,2^

We concur with Li and Tan regarding automatic segmentation in retinal thickness measurements. The central macula can now be evaluated using OCT automated segmentation mapping, similar to retinal measurements, and choroidal thickness can also be measured with Early Treatment Diabetic Retinopathy Study subfield segmentations.^3^ However, using the line-field or wide-field modalities of swept source OCT may also better evaluate the anatomical structures of the choroid and retina in future studies.^4,5^

Best Regards,

## References

[1] Han JY, Lee DC, Lee SY (2018). Horizontal Extraocular Muscle and Scleral Anatomy in Children: A Swept-Source Anterior Segment Optical Coherence Tomography Study. Korean J Ophthalmol..

[2] Oliveira C, Tello C, Liebmann J, Ritch R (2006). Central corneal thickness is not related to anterior scleral thickness or axial length. J Glaucoma..

[3] Tan CS, Cheong KX, Lim LW, Li KZ (2014). Topographic variation of choroidal and retinal thicknesses at the macula in healthy adults. Br J Ophthalmol..

[4] Fechtig DJ, Grajciar B, Schmoll T, Blatter C, Werkmeister RM, Drexler W, Leitgeb RA (2015). Line-field parallel swept source MHz OCT for structural and functional retinal imaging. Biomed Opt Express..

[5] Hong EH, Shin YU, Kang MH, Cho H, Seong M (2018). Wide scan imaging with swept-source optical coherent tomography for glaucoma diagnosis. PLoS One..
